# Hosting Infection: Experimental Models to Assay *Candida* Virulence

**DOI:** 10.1155/2012/363764

**Published:** 2011-12-22

**Authors:** Donna M. MacCallum

**Affiliations:** Aberdeen Fungal Group, School of Medical Sciences, Institute of Medical Sciences, University of Aberdeen, Foresterhill, Aberdeen, AB25 2ZD, UK

## Abstract

Although normally commensals in humans, *Candida albicans*, *Candida tropicalis*, *Candida parapsilosis*, *Candida glabrata,* and *Candida krusei* are capable of causing opportunistic infections in individuals with altered physiological and/or immunological responses. These fungal species are linked with a variety of infections, including oral, vaginal, gastrointestinal, and systemic infections, with *C. albicans* the major cause of infection. To assess the ability of different *Candida* species and strains to cause infection and disease requires the use of experimental infection models. This paper discusses the mucosal and systemic models of infection available to assay *Candida* virulence and gives examples of some of the knowledge that has been gained to date from these models.

## 1. *Candida* and Man

### 1.1. Carriage of *Candida* Species

In healthy individuals *Candida* species are harmless members of the normal gastrointestinal (GI), oral, and vaginal microbial flora. It is assumed that everyone carries *Candida* in their GI tract (reviewed in [1]), with *C. albicans* the species most frequently identified in faecal sampling, representing 40–70% of isolates [2–4]. Other isolates are usually identified as *C. parapsilosis, C. glabrata, C. tropicalis, *or *C. krusei* [2–4].

In comparison to GI carriage, oral carriage is observed in only ~40% of healthy individuals, with considerable variation found between studies (reviewed in [1]). Higher carriage levels are generally associated with diabetes, cancer, HIV, or denture use (reviewed in [1]). Again, the majority of isolates (~80%) are identified as *C. albicans*, with *C. glabrata* or *C. parapsilosis* making up the remainder [5–9].

Vaginal carriage occurs in an even smaller proportion of the healthy population, with only ~20% of healthy women found to have vaginal *Candida* carriage [10–13]. *C. albicans *is again the most commonly identified species, with *C. glabrata* the only other species usually found [10, 12, 14–17]. 

Therefore, *C. albicans* is the major species found as a commensal in healthy individuals, with four other species, *C. tropicalis*, *C. parapsilosis*, *C. glabrata,* and *C. krusei*, also found.

### 1.2. *Candida* and Disease

*Candida* species, however, have an alternative lifestyle, causing opportunistic infection in hosts with altered physiological or immune response. The infections caused by *Candida* species range from self-limiting, superficial mucosal lesions (commonly referred to as thrush), chronic and/or recurrent mucosal, skin, and nail infections, through to life-threatening invasive or disseminated infection [1, 18–21]. 

In humans, the most common infections caused by *Candida* species are superficial infections of the mucosa, skin, and nails [20–24]. Pseudomembranous oral thrush is common in babies and in the elderly, but is also found in HIV-positive individuals and cancer patients (reviewed in [1, 25]). Denture stomatitis is also a significant infection, occurring in approximately 60% of denture wearers [26, 27]. In oral candidiasis most infections are caused by *C. albicans* (58%), with the remainder caused by *C. parapsilosis, C. tropicalis, C. glabrata, *and* C. krusei *[28, 29]. 

Vaginal candidiasis, or thrush, another form of superficial infection, affects approximately 75% of women of child-bearing age [30, 31]. *C. albicans* is most commonly isolated, with *C. glabrata* also found, but at a lower frequency [17, 30, 32–35], reflecting the species normally carried in the vulvovaginal area. 

An additional form of candidiasis involving the mucous membranes, as well as the skin and nails, is chronic mucocutaneous candidiasis. Unlike other forms of candidiasis, there is evidence that this condition can be inherited or is associated with thymoma, with almost every infection caused by *C. albicans* [20–24, 36].

The most serious infections caused by *Candida* species, however, are invasive or disseminated infections. *Candida* species cause ~11% of all bloodstream infections and 20% of those occurring in the ICU population [37–39]. However, in comparison to bacterial infections occurring in the same patient population, these infections are much more serious as mortality rates remain high (~45%) [1, 40]. This is due, in part, to diagnostic difficulties and limited antifungal therapies. Invasive infections occur in those patients who are already seriously ill, with major risk factors including admission to ICU, surgery (especially abdominal surgery), and neutropenia (reviewed in [1]). The five *Candida* species commonly isolated from the human GI tract are also responsible for 90% of invasive *Candida* infections [1, 41]. Geographical variations in the epidemiology of these infections do occur, with *C. tropicalis* the most common cause of invasive *Candida* infection in both India and Singapore [42–44]. In addition, in patients with haematological malignancies and in young children and babies, there is increased incidence of *C. tropicalis* and *C. parapsilosis* [45–49].

Patients with invasive *Candida* infection usually present with clinical symptoms similar to those associated with invasive bacterial infection and can eventually develop sepsis [50]. From autopsy reports, it is evident that the lungs and the kidneys are the organs most commonly affected, with fungal lesions also found in the heart, liver, and spleen [51–55]. Infection most likely originates from the GI tract, as the majority of invasive infections show GI involvement (oesophagus, stomach, and intestines) [51, 53] and *Candida* isolates from the bloodstream are identical, or closely related, to isolates from nonsterile sites of the same patient [56].

Increasing numbers of patients suffering immunosuppression and undergoing invasive treatments, for example, for cancers and organ transplants, mean that there is an ever-increasing population at risk of invasive fungal infection. With a medical need for the development of new and more efficient diagnostics and therapies for fungal infection, we need a better understanding of *Candida* pathogenesis, that is, how do the major *Candida* species cause opportunistic infections?

## 2. Experimental Models of *Candida* Infection

Experimental infection models allow disease development to be followed from the moment that fungal cells are introduced into the host. To be a good model, a model should be reproducible, relatively easy to set up, and should reproduce the major clinical symptoms seen in the human disease. It is also an added advantage if the model is cost effective. Models which satisfy these conditions allow further in-depth investigation of *Candida* virulence to be carried out and, subsequently, allow inferences about *Candida* virulence in human disease to be made. 

Although a great deal of preliminary research on virulence can be carried out by laboratory experiment, infection modelling requires the involvement of a host organism. It is only in a whole organism that the complex host-fungus interactions that determine whether or not disease will occur can be investigated. Although larger animals have been used to study *Candida* infections, for example, macaques [57, 58], piglets [59], rabbits [60–62], and guinea pigs [63, 64], the majority of *Candida* virulence studies use rodent infection models. This is due to economic factors, ease of handling, and the availability of genetically modified mouse strains, which allow human genetic conditions to be mimicked. 

In this paper, experimental animal models that have been developed for *Candida* virulence assays are discussed. It should be noted that the majority of models focus on *C. albicans *as this is the major species associated with human *Candida* infections.

### 2.1. Mucosal Infection Models

To model *Candida* oral and vaginal infections, mucosal models have been developed mainly in rats and mice. The procedures used in rats and mice are generally similar. However, the larger animal has the added advantage that denture-associated fungal biofilms formation can also be studied in a host [65]. Establishment of infection at mucosal sites generally requires treatment with immunosuppressive agents, oestrogen, or antibiotics prior to infection, or the use of germ-free animals [66–68]. However, the nude (*Foxn1^nu^*) mouse model of oral infection allows infection to be established without any immunosuppression or other pretreatment [69]. Greater detail can be found in more extensive reviews of these infection models [67, 68, 70, 71].

In order to assess virulence in mice using the oral infection model, mice are routinely pretreated with corticosteroids and *Candida* cells are administered into the oral cavity of anaesthetised animals either by applying a *Candida*-soaked cotton bud under the tongue or by applying the inoculum directly onto the teeth, gums, and oral cavity [67, 70, 72]. Virulence in this model is usually determined by fungal organ burden and histopathology. 

Both rat and mouse models have been used to compare the virulence of *C. albicans* mutant strains and also clinical isolates [73–77]. Using these models, *C. albicans* mutant strains which are unable to switch between the yeast and hyphal growth forms were found to be unable to cause oral infection, demonstrating a requirement for yeast-hypha switching in oral infection [75]. In addition, protein kinase Ck2 was also shown to be required for oropharyngeal *C. albicans* infections [77].

Mouse and rat models have also been developed to assay *Candida* virulence in vaginal infection. In these models the rodents are maintained in oestrus in order to maintain colonisation and infection, which probably mimics pregnancy-associated candidiasis [78–81]. In rats, this generally involves surgery to remove the ovaries, with subsequent administration of oestrogen [81]. Recently, however, a new rat model has been developed, similar to the mouse model, where oestrus is maintained merely through administration of oestrogen [82], which will increase the ease of setting up the infection model. Immunosuppression of the host can also prolong colonisation by *Candida* species [83]. These models allow us to examine single vaginitis episodes; however, a satisfactory model of recurrent, chronic vaginitis is not yet available.

The virulence of *C. albicans* clinical isolates has been compared in rodent vaginitis models, demonstrating that isolates have varying capacities to cause disease [84, 85]. This model has also been used to assess virulence of genetically modified *C. albicans* mutants [85–87]. 

In addition to assessing *C. albicans* virulence, this model can be used to examine virulence of other *Candida* species. As *C. glabrata* is also associated with human vaginal infection, researchers have used the rat vaginitis model to evaluate the virulence of a *C. glabrata* petite mutant, discovering than the mutant was more virulent that the parental strain [88]. In addition, *C. parapsilosis* isolates have also been assessed for their ability to cause vaginal infection in the rat model [80]. In this study only a single isolate, recently obtained from a woman with active vaginal infection, was capable of initiating infection [80].

A major development in *Candida* virulence testing at mucosal surfaces occurred recently with the development of a concurrent oral and vaginal infection model by Rahman et al. [72]. This mouse model allows both oral and vaginal infections to be initiated in the same host, greatly reducing the numbers of animals required for these virulence assays. A comparison of the virulence of three different *C. albicans* isolates in this model clearly demonstrated that *C. albicans* isolates were not equally virulent, with obvious differences in their ability to initiate mucosal infections [72]. 

### 2.2. Invasive Infection Models

Mouse models of invasive fungal infection have been the most popular methods to assess *Candida* virulence up until the present day, although assays have also been carried out in rabbits, guinea pigs, and rats also used in some studies. There are two major models of *Candida* invasive infection, the intravenous (IV) challenge model and the gastrointestinal (GI) colonisation with subsequent dissemination model. These models were recently reviewed [89].

#### 2.2.1. Intravenous Challenge Model

The mouse IV challenge model has been used to study *Candida* virulence since the 1960s and is both well characterised and reproducible [90–92]. *Candida* cells are injected directly into the lateral tail vein, bypassing any requirement of the fungus to cross epithelial and endothelial barriers to gain entry into the bloodstream. In this mouse model, which is similar to human invasive infection occurring with catheter involvement, fungal cells are found in all organs, but disease progresses only in the kidneys and brain, which depends upon inoculum level and mouse strain [91–93]. Sepsis develops as invasive disease progresses, which eventually leads to the death of the mouse [92, 94, 95]. 

In these models of *Candida* invasive infection, virulence is determined by monitoring survival of infected mice and/or by quantifying fungal organ burdens at predetermined times after infection. Drug treatments can also be administered to the host to allow host conditions to be mimicked, for example, immunosuppression [88, 96–110] or diabetes [99], with greater *Candida* virulence in both of these treatments.

Using immunocompetent mice, the IV challenge model has been used to compare the virulence of different *Candida* species [97–99, 107, 111–114]. *C. albicans* is clearly the most virulent species [97, 98, 111, 112, 114], followed closely by *C. tropicalis* [97, 98, 111, 112, 114]. In contrast, *C. krusei* and *C. parapsilosis* were unable to kill the infected animals, even at high inoculum levels, and fungi were eventually cleared from the host [98, 111, 114]. 

In immunosuppressed mice, *C. tropicalis* showed greater virulence, with disease progressing in the kidneys, rather than infection being controlled which occurs in immunocompetent mice [96, 98, 99, 107, 115]. *C. parapsilosis* and *C. krusei* remained unable to initiate progressive infections, even with addition of immunosuppressive treatments [98, 107], although administration of a very high inoculum potentially allows some *C. parapsilosis* isolates to initiate disease [108, 110]. 

Within each *Candida* species, clinical isolates were found to show considerable virulence differences in the IV challenge model. This was true for *C. albicans* [97, 107, 116, 117], *C. tropicalis* [97, 99, 112, 115, 118], and *C. parapsilosis* [108, 119], with some isolates unable to initiate invasive infections. This raises questions as to whether virulence results found for a single strain or isolate are representative of the entire species. This could be of particular importance for *C. albicans* studies where the vast majority of gene disruption studies have been carried out in a single strain, SC5314, background. 

Numerous studies have evaluated *C. tropicalis* clinical isolate virulence differences; however, there are very few studies published on the virulence of genetically modified *C. tropicalis* strains. One study which has been published was able to demonstrate that a secreted acid protease was required for full virulence of *C. tropicalis* in immunocompetent mice [120]. In contrast to *C. tropicalis*, vast numbers of studies have been published on the virulence of *C. albicans *mutants, with over 200 genes identified as contributing to the *C. albicans* virulence in this model (reviewed in [89]). 

*C. glabrata* behaves very differently from the other *Candida* species in the mouse model of invasive infection. Although *C. glabrata* is maintained, or tolerated, at high levels in the kidneys of immunocompetent mice, the mice did not die and there was little inflammation associated with the fungal cells [113, 114]. Immunosuppression appears to increase virulence of *C. glabrata* in terms of higher fungal organ burdens, but mouse survival is only increased in some *C. glabrata* infections [100, 103–106]. However, because immunosuppression may allow invasive disease to develop in *C. glabrata*-infected mice, these treatments have been added to an infection model used in some studies to compare the virulence of genetically modified *C. glabrata*, with fungal burdens used as the virulence estimate [88, 101, 102, 105]. The immunosuppressed mouse infection model has demonstrated the importance of hypertonic stress responses, the cell wall integrity pathway, and nitrogen starvation responses in *C. glabrata* virulence [103, 104, 106]. In addition, this model has identified a petite mutant, strains expressing hyperactive alleles of the transcription factor gene *PDR1* and the *ace2* null mutant as being more virulent than their parent strains [88, 105, 121]. However, it should be noted that the hypervirulent phenotype of the *C. glabrata ace2* null was completely lost in immunocompetent mice [122]. In other virulence experiments in immunocompetent mice, where virulence was determined from fungal organ burdens at day 7 after infection, researchers were able to demonstrate that the cell wall integrity pathway [123, 124] and oxidative stress response [125], as well as the transcription factor Pdr1p and some of the genes that it regulates [101, 121], contribute to *C. glabrata* virulence.

#### 2.2.2. Gastrointestinal Colonisation and Dissemination Model

Gastrointestinal models can either be set up in neonatal or adult mice. Intragastric infection of neonatal mice leads to persistent colonisation, without any requirement for pretreatment of the mice. However, to obtain colonisation of adult mice, the natural mouse gastrointestinal flora must first be removed by treatment with broad spectrum antibiotics. Adult mice can either be infected by gavage (intragastrically) or orally via their chow or drinking water. Subsequent treatment of *Candida* colonised mice with immunosuppressants and/or drugs which damage the gut wall allow fungal dissemination to occur (reviewed in [70, 126]).

In the gastrointestinal models fungal colonisation is highest in the stomach, caecum, and small intestine [107, 127–129], reflecting some of the clinical findings seen in human invasive infection. During the model, persistent colonisation is routinely monitored by noninvasive faecal fungal counts, and after dissemination *Candida* cells can be cultured from the liver, kidneys, and spleen [128–130]. However, differences may be seen between mouse strains [131].

This murine model is believed to be a more accurate reflection of the events occurring in the human patient, with broad spectrum antibiotics allowing fungal overgrowth and later invasive therapies causing mucosal damage. Mucosal damage then allows *Candida* to enter the bloodstream and disseminate to the internal organs. In the mouse, similar to human patients, there is increased animal-to-animal variation compared to the intravenous challenge model, requiring higher numbers of animals per group to obtain statistically significant results [128–130]. 

Comparison of *Candida* species virulence in this model demonstrated that *C. parapsilosis* had lower virulence compared to *C. albicans* and *C. tropicalis,* as there was little evidence of dissemination from the gut [107, 132]. However, *C. parapsilosis* was successful in establishing persistent colonisation of the GI tract [107]. In separate studies, *C. tropicalis* appeared to be more virulent than *C. albicans* in the gastrointestinal model, with greater dissemination to the internal organs [133, 134] and higher mortality rates [97, 134]. However, given the levels of variation observed in other models for the virulence of strains of different *Candida* species, further isolates will require to be assayed before a definitive conclusion on the relative virulence of the two species can be made. 

To date, only a limited number of *C. albicans* mutant strains have been tested in the gastrointestinal colonisation and dissemination infection model, with only 6 mutants identified so far as contributing to virulence [89, 135]. However, this model has demonstrated that a constitutively filamentous *C. albicans* mutant was unable to disseminate, suggesting that the ability to switch between morphological forms may be more important for dissemination [136]. 

*C. glabrata* also behaved differently from the other four major *Candida* species in this model, being unable to colonise the oesophageal tissue in the neonatal mouse gastrointestinal colonisation and dissemination model [137]. Again, there was little host inflammatory response to *C. glabrata* [137], suggesting that *C. glabrata* virulence mechanisms may be quite different from those of the other species studied.

## 3. Beyond the Genome: Challenges of *Candida* Virulence Testing in the Postgenomic Era

The genome sequences of *C. albicans*, *C. glabrata*, *C. tropicalis,* and *C. parapsilosis* are now available [138, 139], encouraging the creation of large-scale mutant libraries. The challenge comes, however, when these large libraries are to be screened for genes involved in fungal virulence, with logistical, financial, and ethical issues to be considered. 

In library screening programmes carried out to date different virulence testing strategies have been taken. Noble et al. [140] used signature-tagged mutagenesis to allow pools of mutants to be assayed in small numbers of animals, significantly reducing the animal numbers required for testing. By contrast, in order to screen a library of 177 *C. albicans* strains for altered virulence, Becker et al. [141] assayed each strain in 15 mice. From these two examples it is clear that traditional testing methods can lead to large numbers of mice being required to assay virulence. However, researchers have recently begun to address the issues of virulence testing large numbers of *Candida* strains by developing a range of minihosts, which are mainly based on invertebrate hosts. 

Minihosts may not initially appear relevant to the human disease, but these hosts do possess an innate immune system and this is known to be critical in the development of *Candida* infections [142]. However, many of the minihosts do not possess an adaptive immune system, which may limit their usefulness. In addition, the majority of invertebrate models have the disadvantage that they must be kept at temperatures below normal human body temperature, with the exception of *Galleria* which can be incubated at 37°C. Potentially, incubation at lower temperatures may induce physiological changes in the fungus, affecting host-fungus interactions during disease development.

### 3.1. Wax Moth and Silk Worm Larval Models

The first minihost model developed for *Candida* virulence testing was the *Galleria mellonella* (wax moth) larval model [143]. In this model fungi are injected into larvae, via a proleg, and survival is monitored over a short time period. The model is relatively cheap and has the added advantage that large numbers of larvae can be infected with each mutant strain, increasing the statistical power of the assay. The *Galleria* model has been successfully used to model *C. albicans* virulence, with results roughly similar to those found in mouse infection models [143–146]. A similar model has also been developed using the silk worm (*Bombyx mori*) [147, 148]. Both *C. albicans* and *C. tropicalis* are capable of killing silk worm larvae within two days [148], and *C. albicans* virulence differences were shown to correlate with results previously found in a mouse model [147].

### 3.2. *Drosophila melanogaster*

The fruit fly, *Drosophila melanogaster*, has also been used to assay *Candida* virulence [149–152]. The susceptibility of wild-type *D. melanogaster* continues to be debated; however, both Toll- and Spätzle-deficient fruit flies are susceptible to infection by *Candida* species when fungi are injected directly into the thorax [149–151]. Again, *D. melanogaster* models also have the advantage that large numbers of flies (>30 flies) can be infected with each *Candida* strain, increasing the statistical power of the assay. 

In fruit flies, *C. albicans* was shown to be more virulent than other *Candida* species, confirming the results found in mammalian models (see above; [149]). In addition, virulence results for *C. albicans* clinical isolates and mutants were broadly similar to those found in the mouse systemic model [149–151]. However, differences do occur. In the fruit fly, CO_2_ sensing is important for virulence, but this was not the case in the mouse IV challenge model [153]. This model has already been successfully used to screen a *C. albicans* mutant library, identifying Cas5, a transcription factor involved in cell wall integrity, as being required for full virulence [154]. 

In addition to the systemic *D. melanogaster* infection model, a new gastrointestinal infection model has also been developed recently, which should provide new options for virulence screening in a gastrointestinal model [152]. 

### 3.3. *Caenorhabditis elegans*

In addition to fly and larval models, the nematode *Caenorhabditis elegans *has also been evaluated as an infection model for assaying *Candida* virulence [155]. This model is particularly suited to high-throughput screening, as the *Candida* cells are fed to the nematodes in their food and assays are carried out in multi-well plates. This model has also been used successfully to screen a *C. albicans* transcription factor mutant library, allowing identification of transcription factor genes involved in hypha formation [155]. 

### 3.4. A Vertebrate Minihost: Zebrafish *(Danio rerio)*

Zebrafish are the first vertebrate minihost model developed for virulence testing of *Candida*. This organism has the added advantage of having both innate and adaptive immune systems [156], and methods are also available to allow fish gene expression to be manipulated to mimic human genetic conditions [157].

The first virulence assay developed in zebrafish involved intraperitoneal injection of *C. albicans* into 7-month-old zebrafish [158]. In this model, similar to mouse models, progressive infection depends upon dose and is associated with increased proinflammatory gene expression. This model also allows increased group sizes, with group sizes of 20 fish being used to date. Using this model, researchers demonstrated that a clinical isolate with reduced virulence in a mouse model also showed reduced virulence in this model [158]. In addition, a *C. albicans* mutant (*efg1/cph1*) known to have attenuated virulence due to filamentation defects also had reduced virulence in this model [159, 160]. Of greater interest was the finding that, although these mutants were unable to form filaments *in vitro*, they clearly formed filaments when growing within fish. This model also allows interactions between zebrafish immune cells and *Candida* cells to be imaged, which will be made even easier in the future with the development of the new transparent adult (*casper*) zebrafish [161]. 

A second zebrafish infection model has also been described, where each fish larva (36 h after fertilization) is infected directly into the hindbrain ventricle with approximately 10 fungal cells [162]. In this model the *C. albicans efg1/cph1* mutant again demonstrated attenuated virulence, similar to results found in the mouse IV challenge model [162]. 

There are, however, disadvantages to the zebrafish infection models. One of the major drawbacks of this model, in common with the majority of other minihosts, is that the fish need to be kept at 28-29°C, which does not allow accurate mimicking of human infection. 

## 4. Assaying Virulence in Experimental Models: Final Considerations

There are some important points to remember when evaluating *Candida* virulence in experimental infections. The first concerns the *Candida* species of interest. Although *C. albicans, C. tropicalis, C. glabrata, *and* C. krusei *are all associated with human carriage and infection, they are not natural mouse commensals or pathogens [163]. As such, there may be different interactions occurring between the fungus and the two different host species. This is of particular relevance when considering *C. glabrata* and its inability to initiate disseminated infection in the IV challenge models, especially when we know that *C. glabrata* can cause lethal infections in severely ill humans [164].

The second point to consider is that, although the immune systems of mice and men are similar, there are differences that could affect how the host and fungus interact [165–168]. Of particular relevance to *Candida* infections are differences in proportions of neutrophils and lymphocytes in the blood, complement receptor expression, and T-cell differentiation, to name but a few (reviewed in [168]). In addition, different mouse strains show differing susceptibility to infection, which could potentially alter virulence results [93, 169–172].

The third point to consider is which model should be used to evaluate *Candida* virulence. Some *C. albicans* isolates exhibit virulence differences depending upon the infection model being used [72, 134, 173]. A good example is the *C. albicans* genome sequenced strain SC5314. In the IV challenge model, SC5314 is one of the most virulent *C. albicans* isolates, causing lethal infection in a relatively short time [92, 116]; however, in a vaginal infection model, SC5314 is a very poor coloniser of the vaginal mucosa [72]. In addition, a nongerminative *C. albicans* strain [173] and a *ura3 *minus *C. albicans* strain [174], both of which were attenuated in systemic infection models [173, 175–177], successfully established mucosal infections [173, 174]. 

Only careful consideration of the above points will allow the *Candida* researcher to select the appropriate experimental *Candida* infection model to answer a particular research question. These models remain essential for increasing our understanding of fungal pathogenesis since both fungal attributes and host responses are known to contribute to the development of clinical disease.
